# Assessing the individual relationships between physical test improvements and external load match parameters in male professional football players—a brief report

**DOI:** 10.3389/fspor.2024.1367894

**Published:** 2024-04-12

**Authors:** Per Thomas Byrkjedal, Thomas Bjørnsen, Live Steinnes Luteberget, Andreas Ivarsson, Matt Spencer

**Affiliations:** ^1^ Department of Sport Science and Physical Education, University of Agder, Kristiansand, Norway; ^2^ Department of Physical Performance, Norwegian School of Sport Sciences, Oslo, Norway; ^3^ School of Health and Welfare, Halmstad University, Halmstad, Sweden

**Keywords:** team sports, GPS, athlete monitoring, player development, performance

## Abstract

**Purpose:**

This study aimed to explore whether a meaningful improvement in physical performance following an in-season strength training intervention can be related to external load match parameters at an individual level in professional male football players.

**Methods:**

Eight male professional football players (25.4 ± 3.1 years, 184.1 ± 3.4 cm, 79.3 ± 2.2 kg) completed a 10-week strength intervention period in addition to football-specific training and matches. Commonly used physical and external load measures were assessed before and after intervention. Physical performance improvements had to exceed the measurement’s typical error and the smallest worthwhile difference (SWD) to be considered meaningful. External load match parameters were assessed before and after the intervention period using SWD and non-overlap of all pairs (NAP) analysis. A Bayesian pairwise correlation analysis was performed to evaluate relationships between changes in physical performance and external load match parameters.

**Results:**

Three players displayed meaningful improvements in two to five physical performance measures. However, positive changes exceeding the SWD and positive effects in NAP results were observed for all players in external load match parameters. Kendall's tau correlation analysis showed evidence (base factor >3) for only one correlation (maximum speed − decelerations, *τ* = −0.62) between the changes in physical performance and external load measures, while the remaining comparisons exhibited no relation.

**Conclusions:**

The findings suggest that improvements in physical performance may not necessarily translate to improvements in external load match parameters. Further research, with larger sample sizes, is needed to understand potential mechanisms between acute and chronic physical performance changes and football external load parameters during training and matches.

## 1 Introduction

Coaches and practitioners may interpret improvements in the physical capacity of fitness tests as coinciding with improvements in physical match performance based on the assumption of a causal relationship between these variables, with little evidence of the construct validity (e.g., dose–response relationship) (1). Well-developed physical performance is indeed important for football-specific performance. However, generic measures of physical performance are influenced by numerous factors, including reliability and validity, which must be considered whenever interpreting changes in physical performance (2, 3). For example, to minimize the impact of extraneous factors, it is imperative to conduct physical testing in controlled environments with an understanding of the equipment's inherent measurement errors. For example, common physical performance measures, such as 10- and 30-m linear sprint time, maximum speed, countermovement jump (CMJ), and leg press power, have demonstrated raw and relative (%) typical error (TE) values of 0.03–0.05 s (TE%: ∼1.3), 0.18 m/s (TE%: 1.4), 1.7 cm (TE%: 4.6), and 70 W (TE%: 4.4), respectively (2). Apart from awareness of reliability, determining the meaningfulness of any observed change is an essential aspect of player monitoring and can, as an example, be calculated by estimating the smallest worthwhile difference (SWD) (2–4). Thus, utilizing the TE and SWD may be seen as feasible criteria in determining whether performance improvements or declines should be interpreted as meaningful or not.

In addition to tracking changes in physical performance over time, external load data are commonly used to monitor training and match load in football at a group and individual level (5, 6). Previous research has found strong cross-sectional associations between physical performance and match running performance in football (7, 8), and football-specific training has been shown to improve physical performance (9). Thus, recent research suggests that external load measures can be reflective of the physical performance of players (10). However, physical performance and external load data are known to differ between competitive levels (7), and there is a lack of knowledge on how changes in physical performance are reflected in external load parameters among highly trained players. For example, speed and explosive movements are regarded as essential for football-specific performance (5, 11), and minor performance enhancements in these players may potentially influence the likelihood of success in match-decisive actions (12, 13). In contrast, external load is typically assessed cross-sectionally, and it is currently unknown how changes in physical performance measures impact external load in match play. In addition, when evaluating highly trained players, subtle differences and unique variations within and between players are of utmost importance (12). Consequently, the assessment of players in elite sports necessitates a personalized approach, highlighting the significance of tailoring evaluations to individual needs (11, 14). On the contrary, research has traditionally focused on group assessments when presenting their findings (6, 14).

With the importance of assessing individual responses in both physical test performance and external match load data, this brief report aims to explore whether a meaningful improvement in the physical test performance of players is related to the external load match performance by assessing the individual player response. This brief report is based on data from a strength intervention study by Byrkjedal et al. including a team of male professional football players (15).

## 2 Methods

**Figure 1:**
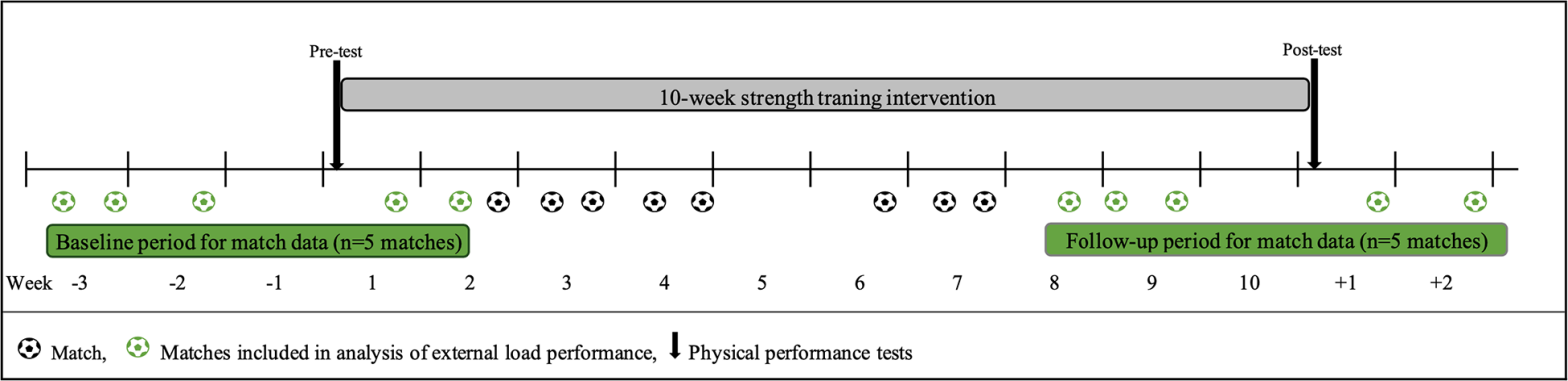
Schematic overview of the study, including specific test points, strength intervention period, and matches played.

This case study originates from a 15-week study where professional footballers underwent a 10-week strength training intervention (15). Physical performance (30-m sprint, CMJ, and leg press power) was measured before and after the intervention, and external load match parameters were monitored for five matches at the start (“baseline”) and at the end (“follow-up”) of the intervention period. An overview of the study period is presented in Figure 1. This report aims to identify meaningful improvements in player's physical test performance and to explore the relationship with changes in external load match parameters. See Byrkjedal et al. (15) for more details on the original study design and data processing.

### 2.1 Subjects

Sixteen outfield players representing a Norwegian second-tier club completed the strength intervention period and were eligible for inclusion in this brief report. However, players had to participate in a minimum of two matches (with ≥60 min playing time per match) in both the baseline and the follow-up period to be included in this brief report. Eight male players (baseline: *n* = 6; follow-up: *n* = 2) were excluded due to lack of participation and/or sufficient playing time. Thus, eight players (25.4 ± 3.1 years, 184.1 ± 3.4 cm, 79.3 ± 2.2 kg) were included for further analysis. Written informed consent was obtained before the study commenced. The study was performed according to the 1975 Declaration of Helsinki, approved by the local ethics committee at the University of Agder, Kristiansand, Norway, and the Norwegian Center for Research Data (approval reference: 464080).

Briefly, physical performance testing before and after the intervention was completed in 1 day using a test battery of 30-m sprint, CMJ, and Keiser leg press. The 30-m sprint test involved two to four maximal sprints with 4-min passive rest, where the best attempt was analyzed. CMJs were completed with two to three sets of three jumps performed 30 s apart, with 2–3-min passive rest in between jump sets. The mean jump height of the two best attempts was analyzed. Lower limb strength and power were assessed using a horizontal pneumatic leg press device with a 10-Repetition maximum protocol (15). Performance enhancements had to exceed raw and relative (%) TE and SWD (2–4) to be considered a meaningful improvement. The same test equipment and protocols as in Lindberg et al. (2) were used, and pre-test results were used to calculate the SWD (3, 4).

Match performance was assessed with a tracking system from Catapult Sports (Vector S7, Firmware 8.10, Catapult Sports, Melbourne, Australia). Ten matches, five in the baseline and five in the follow-up period, were included to investigate the effect in external load match parameters after the intervention period. External load parameters, relative to playing time, included distance per minute, PlayerLoad™, high-speed running (19.8–25.2 km/h; HSR) and sprint running (>25.2 km/h) distance, accelerations, decelerations, and change of directions (summary of movements in the respective direction's with an intensity >2.5 m/s). The sum of these constituted high-intensity events (16).

### 2.2 Statistics

Descriptive results were calculated using Microsoft Excel (version 16.67, Microsoft Corp., Redmond, WA, USA) and are reported as mean ± SD (standard deviation). Differences in external load parameters are reported as mean with 95% upper and lower confidence limits. A non-parametric Bayesian correlation analysis was performed in JASP (Jeffreys's Amazing Statistics Program, version 0.16.1) to investigate the relationship between the physical test performance and external load parameters. The Kendall tau correlations, in combination with Bayes factor (BF) values, were calculated for each comparison. The BF is one method to quantify the likelihood of an alternative hypothesis (H1) compared with the null hypothesis (H0) and is expressed as BF_10_. A BF_10_ >3 was interpreted as evidence supporting the association. For a more comprehensive description and full interpretation of BF_10_, see Byrkjedal et al. (16).

Differences in external load match parameters between the baseline and follow-up periods were analyzed using the SWD, which was calculated as 0.2 of the SD between players at the pre-test/baseline (3), and non-overlap of all pairs (NAP). NAP is a non-parametric technique for measuring the non-overlap or “dominance” for two phases and is a feasible way to interpret individual effects between two periods. Advantages of the NAP are, for example, that it can be applied in distributions that lack normality and all data points collected are included in the analyses. Its disadvantages are that it cannot be used to evaluate trends or serial dependency. For a more thorough explanation of NAP and its application, see the study by Parker and Vannest (17). Effect sizes for NAP values were interpreted according to previous recommendations: 0–0.65 = weak effects, 0.66–0.92 = moderate effects, and 0.93–1.0 = large or strong effects (17).

## 3 Results

**Figure 2:**
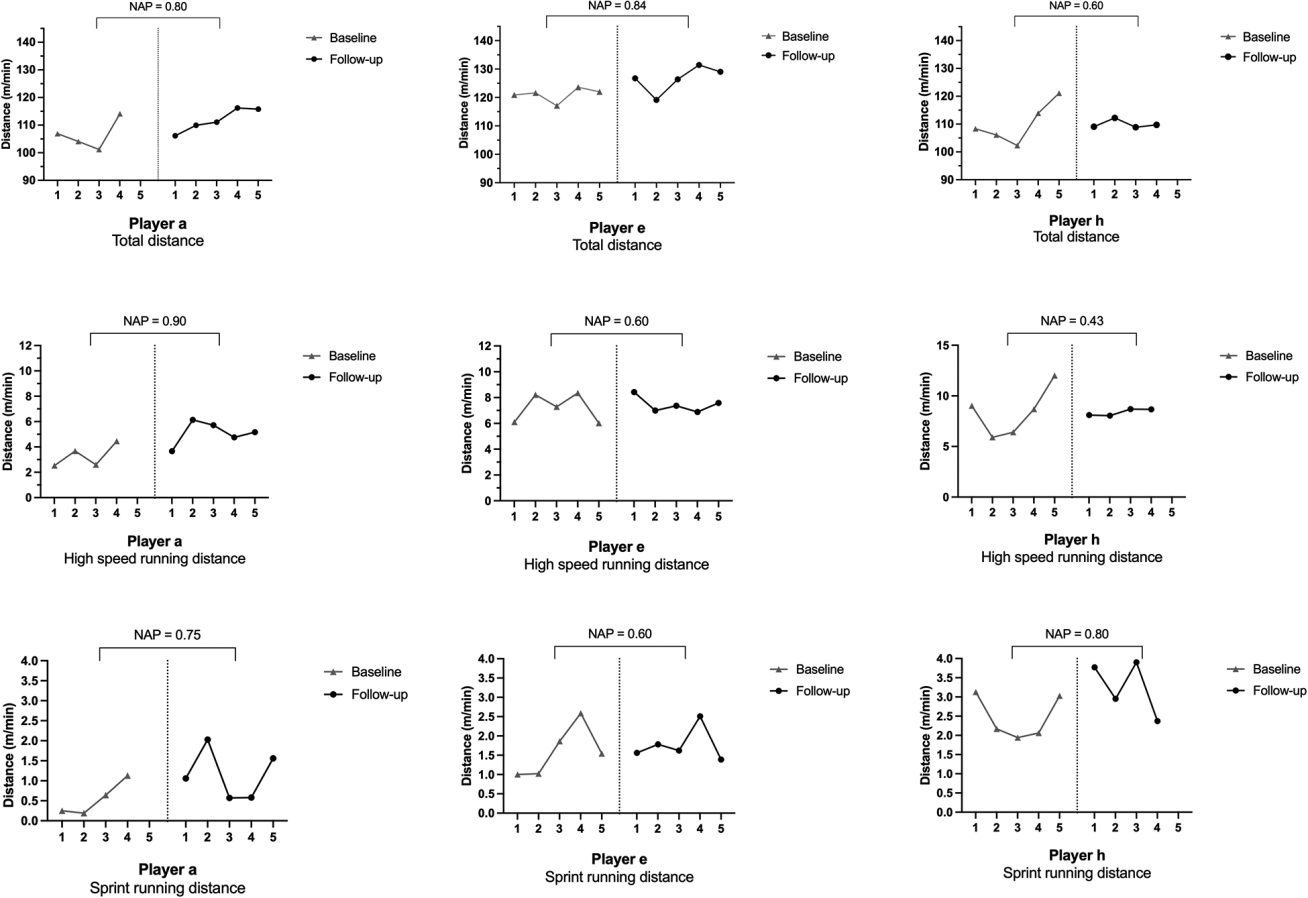
Non-overlap of all pairs analysis results for total distance, high-speed running distance, and sprint running distance for players with a meaningful improvement in physical performance after the strength intervention period.

Results from the pre- and post-intervention period and changes in physical test performance and external load match parameters are presented in Table 1. Kendall's tau correlations between changes in physical test performance and external load are presented in Table 2. Three players exhibited physical test improvements exceeding the SWD, TE, and TE%, and their individual NAP effects in the three most common external load match parameters (total, high-intensity running, and sprint running distance) (5) are presented in Figure 2. Individual figures and NAP effects across all variables for all eight players are available in the Supplementary material.

**Table 1:** Individual results and change from pre-test/baseline to post-test/follow-up in physical test performance and external load match parameters.

	Physical performance					External load								
	10-m	30-m	Max speed	CMJ	Pmax	TD	Peak speed	Player Load	HSR	SPR	HIE	Acc	Dec	CoD
	s	s	m/s	cm	W	m/min	m/s	au/min	m/min	m/min	nr/min	>2.5 nr/min	>2.5 nr/min	>2.5 nr/min
Pre-test/baseline period														
Player a (*n* = 4)	1.60	4.04	8.64	41.5	1,534	106.6 ± 5.5	7.96 ± 0.58	10.6 ± 0.8	3.31 ± 0.92	0.55 ± 0.44	1.03 ± 0.06	0.21 ± 0.04	0.21 ± 0.03	0.61 ± 0.02
Player b (*n* = 5)	1.65	4.27	7.97	27.4	1,165	132.4 ± 7.0	7.56 ± 0.20	13.7 ± 1.2	7.92 ± 1.77	0.66 ± 0.39	1.01 ± 0.14	0.25 ± 0.06	0.19 ± 0.04	0.58 ± 0.09
Player c (*n* = 5)	1.44	3.71	9.42	46.9	1,164	112.2 ± 4.1	8.74 ± 0.39	12.0 ± 0.3	6.62 ± 1.48	1.90 ± 0.79	0.93 ± 0.13	0.21 ± 0.03	0.24 ± 0.03	0.47 ± 0.08
Player d (*n* = 4)	1.49	3.85	9.06	44.0	1,902	100.4 ± 4.1	8.01 ± 0.39	9.7 ± 0.5	2.28 ± 0.84	0.51 ± 0.35	1.44 ± 0.07	0.41 ± 0.10	0.22 ± 0.04	0.81 ± 0.09
Player e (*n* = 5)	1.49	3.80	9.36	47.5	2,098	121.0 ± 2.4	8.29 ± 0.24	12.9 ± 0.3	7.19 ± 1.12	1.60 ± 0.66	1.21 ± 0.13	0.28 ± 0.05	0.27 ± 0.08	0.66 ± 0.07
Player f (*n* = 3)	1.54	3.93	8.77	42.7	1,597	128.6 ± 6.1	8.25 ± 0.49	13.3 ± 0.8	7.94 ± 1.55	1.98 ± 1.24	1.26 ± 0.04	0.23 ± 0.03	0.35 ± 0.05	0.68 ± 0.08
Player g (*n* = 2)	1.46	3.79	9.09	38.7	1,719	127.0 ± 8.1	8.33 ± 0.21	11.3 ± 1.3	7.61 ± 1.33	1.01 ± 0.19	1.59 ± 0.39	0.36 ± 0.09	0.33 ± 0.11	0.90 ± 0.19
Player h (*n* = 5)	1.52	3.92	8.87	39.0	1,673	110.3 ± 7.3	8.32 ± 0.34	10.4 ± 0.9	8.40 ± 2.43	2.47 ± 0.57	1.51 ± 0.19	0.24 ± 0.02	0.31 ± 0.09	0.96 ± 0.11
Post-test/follow-up period														
Player a (*n* = 5)	1.53	3.88	9.06	49.4	1,599	111.8 ± 4.2	8.58 ± 0.55	11.2 ± 0.6	5.08 ± 0.95	1.16 ± 0.63	1.17 ± 0.07	0.30 ± 0.05	0.18 ± 0.02	0.70 ± 0.10
Player b (*n* = 3)	1.67	4.29	7.94	28.7	1,035	130.4 ± 3.0	7.70 ± 0.40	12.5 ± 0.6	8.72 ± 0.79	0.90 ± 0.47	1.04 ± 0.15	0.20 ± 0.06	0.22 ± 0.04	0.61 ± 0.20
Player c (*n* = 5)	1.47	3.78	9.35	43.8	1,105	115.3 ± 3.5	8.59 ± 0.47	12.1 ± 0.4	8.30 ± 1.00	2.50 ± 0.49	1.19 ± 0.20	0.27^a^ ± 0.03	0.26 ± 0.06	0.65 ± 0.18
Player d (*n* = 5)	1.51	3.89	9.14	42.8	1,764	107.8^a^ ± 3.6	8.31 ± 0.26	10.4 ± 0.4	4.93^a^ ± 1.01	0.93 ± 0.44	1.30 ± 0.16	0.39 ± 0.08	0.21 ± 0.05	0.69 ± 0.13
Player e (*n* = 5)	1.46	3.72	9.60	55.7	2,267	126.5 ± 4.6	8.57 ± 0.56	13.3 ± 0.4	7.45 ± 0.61	1.77 ± 0.43	1.42 ± 0.11	0.33 ± 0.07	0.25 ± 0.06	0.84^a^ ± 0.10
Player f (*n* = 5)	n/a	3.99	n/a	39.6	1,478	130.3 ± 3.2	8.26 ± 0.37	13.4 ± 0.3	7.93 ± 1.22	1.67 ± 0.55	1.77^a^ ± 0.12	0.34^a^ ± 0.04	0.42 ± 0.06	1.00^a^ ± 0.15
Player g (*n* = 4)	1.46	3.79	9.06	38.9	1,683	126.6 ± 3.8	8.81^a^ ± 0.24	11.7 ± 0.8	8.73 ± 0.92	2.08^a^ ± 0.51	1.39 ± 0.13	0.32 ± 0.07	0.20 ± 0.04	0.86 ± 0.13
Player h (*n* = 4)	1.52	3.88	9.31	43.9	1,573	110.0 ± 1.6	8.45 ± 0.20	10.2 ± 0.3	8.38 ± 0.35	3.25 ± 0.72	1.30 ± 0.06	0.27 ± 0.01	0.23 ± 0.02	0.81 ± 0.04
Change: pre/baseline period to post/follow-up period														
Player a	0.08*	0.16*	0.42*	7.9*	65	5.3^b^	0.62^b^	0.7^b^	1.77^b^	0.61^b^	0.14^b^	0.08^b^	−0.03	0.09^b^
						−2.4 to 12.9	−0.28 to 1.51	−0.4 to 1.8	0.28 to 3.26	−0.28 to 1.49	0.03 to 0.25	0.01 to 0.15	−0.07 to 0.01	−0.03 to 0.22
Player b	−0.02	−0.02	−0.04	1.3	−130	−2.0	0.14^b^	−1.3	0.80^b^	0.24^b^	0.02	−0.05	0.04^b^	0.03
						−12.1 to 8,6	−0.36 to 0.64	−3.1 to 0.6	−1.90 to 3.50	−0.51 to 0.99	−0.23 to 0.28	−0.15 to 0.06	−0.03 to 0.11	−0.21 to 0.28
Player c	−0.03	−0.07	−0.07	−3.1	−59	3.1^b^	−0.14	0.1	1.68^b^	0.60^b^	0.26^b^	0.06^b^	0.02^b^	0.18^b^
						−3.1 to 11.5	−0.72 to 0.44	−0.4 to 0.6	−0.17 to 3.52	−0.42 to 1.62	0.02 to 0.50	0.01 to 0.11	−0.05 to 0.10	−0.02 to 0.38
Player d	−0.02	−0.04	0.08	−1.2	−138	7.4^b^	0.30^b^	0.7^b^	2.65^b^	0.42^b^	−0.14	−0.02	−0.01	−0.12
						1.4 to 13.7	−0.21 to 0.82	0.1 to 1.3	1.15 to 4.14	−0.23 to 1.06	−0.35 to 0.06	−0.16 to 0.13	−0.08 to 0.07	−0.30 to 0.06
Player e	0.03*	0.08*	0.23*	8.2*	169*	5.5^b^	0.28^b^	0.4^b^	0.25	0.17^b^	0.22^b^	0.05^b^	−0.03	0.19^b^
						0.1 to 10.9	−0.35 to 0.90	−0.1 to 1.0	−1.06 to 1.60	−0.65 to 0.99	0.05 to 0.38	−0.04 to 0.14	−0.13 to 0.07	0.06 to 0.31
Player f	n/a	−0.05	n/a	−3.0	−119	1.7	0.00	0.0	−0.01	−0.32	0.51^b^	0.11^b^	0.07^b^	0.32^b^
						−6.9 to 10.3	−0.74 to 0.75	−1.0 to 1.0	−2.40 to 2.39	−1.82 to 1.19	0.33 to 0.68	0.05 to 0.18	−0.03 to 0.17	0.09 to 0.56
Player g	0.00	0.00	−0.03	0.2	−36	−0.4	0.48^b^	0.4^b^	1.12^b^	1.06^b^	−0.20	−0.04	−0.13	−0.03
						13.0 to 12.2	−0.08 to 1.04	−1.9 to 2.7	−1.39 to 3.63	−0.03 to 2.17	−0.75 to 0.35	−0.21 to 0.14	−0.29 to 0.03	−0.39 to 0.32
Player h	0.00	0.03	0.45*	5.0*	−100	−0.3	0.13^b^	−0.3	−0.03	0.78^b^	−0.20	0.03^b^	−0.08	−0.15
						−9.3 to 8.6	−0.33 to 0.59	−1.3 to 0.81	−2.97 to 2.91	−0.23 to 1.79	−0.44 to 0.03	0.01 to 0.05	−0.19 to 0.03	−0.29 to −0.01

n, number of included matches in the respective periods; Pmax, Max power (W); TD, total distance; AU, arbitrary units; HSR, high-speed running; SPR, sprint running distance; HIE, high-intensity events; Acc, accelerations; Dec, decelerations; CoD, change of directions; N/a: missing data.

Positive change in 10- and 30-m sprint times indicate improved performance from pre to post. Physical performance results are reported with raw data points and raw differences. External load parameters are reported with mean ± SD in the baseline and follow-up period, while changes are reported as mean differences including 95% lower and upper confidence limits.

*Bold text indicates that physical test performance changes were > SWD, raw and relative (%) TE.

^a^ Strong effects in non-overlap of all pair analysis (in follow-up period compared to baseline period).

^b^ >SWD calculated from baseline period results.

**Table 2:** Kendall's tau correlations between changes in physical performance and external load match parameters from pre-test/baseline period to post-test/follow-up period.

	10-m	30-m	Max speed	CMJ	Pmax
TD	0.07 (−0.38 to 0.48)	0.07 (−0.38 to 0.48)	0.14 (−0.33 to 0.53)	0.07 (−0.38 to 0.48)	0.21 (−0.28 to 0.57)
Peak speed	0.22 (−0.27 to 0.58)	0.57 (−0.03 to 0.78)	0.36 (−0.18 to 0.66)	0.43 (−0.13 to 0.70)	0.14 (−0.33 to 0.53)
PlayerLoad™	0.15 (−0.33 to 0.53)	0.21 (−0.28 to 0.57)	0.29 (−0.23 to 0.62)	0.07 (−0.38 to 0.48)	0.36 (−0.18 to 0.62)
HSR	−0.30 (−0.63 to 0.22)	−0.07 (−0.49 to 0.38)	0.00 (−0.43 to 0.43)	−0.21 (−0.57 to 0.28)	−0.07 (−0.48 to 0.38)
SPR	−0.22 (−0.58 to 0.27)	0.14 (−0.33 to 0.53)	0.36 (−0.18 to 0.66)	0.00 (−0.43 to 0.43)	0.14 (−0.33 to 0.53)
HIE	0.15 (−0.32 to 0.53)	−0.26 (−0.60 to 0.25)	−0.47 (−0.73 to 0.10)	−0.11 (−0.50 to 0.35)	0.11 (−0.35 to 0.50)
Acc	0.52 (−0.07 to 0.75)	0.07 (−0.38 to 0.48)	0.00 (−0.43 to 0.43)	−0.07 (−0.48 to 0.38)	0.21 (−0.28 to 0.57)
Dec	−0.04 (−0.46 to 0.40)	−0.40 (−0.69 to 0.15)	−0.62^a^ (−0.80 to −0.01)	−0.33 (−0.65 to 0.20)	−0.33 (−0.65 to 0.20)
CoD	0.30 (−0.21 to 0.63)	−0.14 (−0.53 to 0.33)	−0.50 (−0.74 to 0.08)	0.00 (−0.43 to 0.43)	0.29 (−0.23 to 0.62)

TD, total distance; HSR, high-speed running; SPR, sprint running distance; HIE, high-intensity events; Acc, accelerations; Dec, decelerations; CoD, change of directions; Pmax, maximum power (W).

^a^ BF_10_ > 3. Values in parentheses indicate 95% lower and upper credible intervals.

## 4 Discussion

This study explored the effects in external load match parameters following a meaningful change in physical test performance after an in-season strength intervention including a small sample of professional football players. Our results suggest that a meaningful change in the physical test performance does not directly impact external load match parameters, and we do not observe changes in the physical test performance to be associated with changes in external load match parameters.

When looking at the results (Table 1), three players (a, e, and h) exhibited meaningful physical test improvements. Contrastingly, several other players exhibited strong NAP effects and changes exceeding the SWD, suggesting that meaningful improvements in physical test performance were not consistently reflected in external load match parameters. Indeed, this study was conducted during the in-season period, with a high football-specific focus likely explaining the uniform improvements in external load match parameters.

An external load has been explored as a simple tool to monitor players’ physical fitness in a previous study, and although some parameters were correlated, it was highlighted that the measures may not be sensitive enough to detect small but meaningful alternations in players’ fitness (10). This observation is coherent with our findings. Furthermore, a small range of physical performance improvements complicates the identification of a relationship; nevertheless, such minor improvements may still be important for football-specific performance. Despite cross-sectional assessments demonstrating a relationship between physical performance and external load data across participants (7, 11), our findings suggest that small but meaningful within-subject improvements in physical performance might not affect external load parameters.

Current research emphasizes the large variations within external load match data; therefore, the lack of sensitivity is a huge challenge when attempting to assess associations in changes of potentially associated data such as physical fitness test results (18). It is possible that larger physical performance improvements typically seen after years of practice, for example, from youth academy to senior elite-level players (7, 8, 11), would be necessary to reflect changes in external load data.

Sport-specific performance, such as match play, is a highly complex task, difficult to decipher by fixed moving patterns such as generic physical performance tests or external load parameters (1, 7, 16). The inherent challenge of identifying small but meaningful performance changes is evident even in simple physical performance assessments (1, 2), and with the variation in external load parameters (11, 15), the lack of an association in the current study is not unexpected. However, the importance of physical performance testing or external load monitoring *per se* should not be neglected. While we emphasize the challenges of assuming a causal relationship between them without supportive data (1), both physical performance results and external load data in themselves can be of high value for practitioners in optimizing player performance and development, minimizing the risk of injuries and preparing for competitive performance (5, 7, 11).

Previously (9, 10) and in the current study, external load match data have been included to explore the relationships with physical performance, despite the known challenges with match-to-match variabilities (19) and the influence of contextual factors (20). However, drills, such as small-sided games, have been thoroughly utilized as a way of standardizing gameplay (21). Such drills may represent a feasible measure of players’ performance and should be further explored as a method to standardize the external load demands when exploring the relationships between physical fitness and external load parameters in future studies (6).

## 5 Practical application

Although this dataset has a small sample size, we believe that our findings can serve as a foundation for future studies. In general, we highlight the need to increase the knowledge on how strength training adaptations can impact a variety of football match external load parameters and performance. With no direct link between improvements in physical performance tests and changes in external load match parameters, coaches and practitioners should evaluate the importance of physical and external load monitoring separately and avoid postulating an effect between two measures without supportive data. We emphasize the need for researchers and practitioners to work closely together to better understand and explore how physical performance changes can potentially affect different measures of football-specific parameters.

## 6 Conclusions

Improvements in physical test performance may not necessarily translate to changes in external load match parameters. More research is needed to address and understand the mechanisms between changes in physical performance and how this affects measures of match-related external load performance. Future studies should include larger samples of trained players and a non-strength training control group to further investigate the relationship between changes in physical test performance and measures of external load from both training and match situations.

## Data Availability

The raw data supporting the conclusions of this article will be made available by the authors without undue reservation.
