# Supplementary material for: Assessing the individual relationships between physical test improvements and external load match parameters in male professional football players—a brief report
Source: Front Sports Act Living. 2024 Apr 12;6:1367894. doi: 10.3389/fspor.2024.1367894 (PMC11045924; doi:10.3389/fspor.2024.1367894)
Supplement: Supplementary file 1 [file Datasheet1.pdf]

Supplementary Material

Assessing the individual relationships between physical test improvements and external load match parameters in male professional football players – A brief report

Per Thomas Byrkjedal*, Thomas Bjørnsen, Live Luteberget, Andreas Ivarsson, Matt Spencer

*** Correspondence:** Per Thomas Byrkjedal: per.byrkjedal@gmail.com

1 Supplementary Figures – Individual player NAP graphs

The article includes 8 players (player a-h) and 9 associated data variables; Total distance, peak speed, PlayerLoad^{TM}, high speed running distance (19.8-25.2 km/h), sprint running distance (>25.2 km/h), high intensity events (HIE), accelerations, decelerations and change of directions. A non-overlap of all pair (NAP) analysis was performed to assess the difference in players external match load efforts before (baseline-period) and after (follow-up period) the strength intervention period. Effect sizes for NAP values were interpreted according to previous recommendations by Paker and Vannest (2009): 0–.65 = week effects, .66–.92 = moderate effects, .93–1.0 = large or strong effects.

Parker RI, Vannest K. An Improved Effect Size for Single-Case Research: Nonoverlap of All Pairs. Behavior therapy (2009) 40(4):357-67. doi: 10.1016/j.beth.2008.10.006.

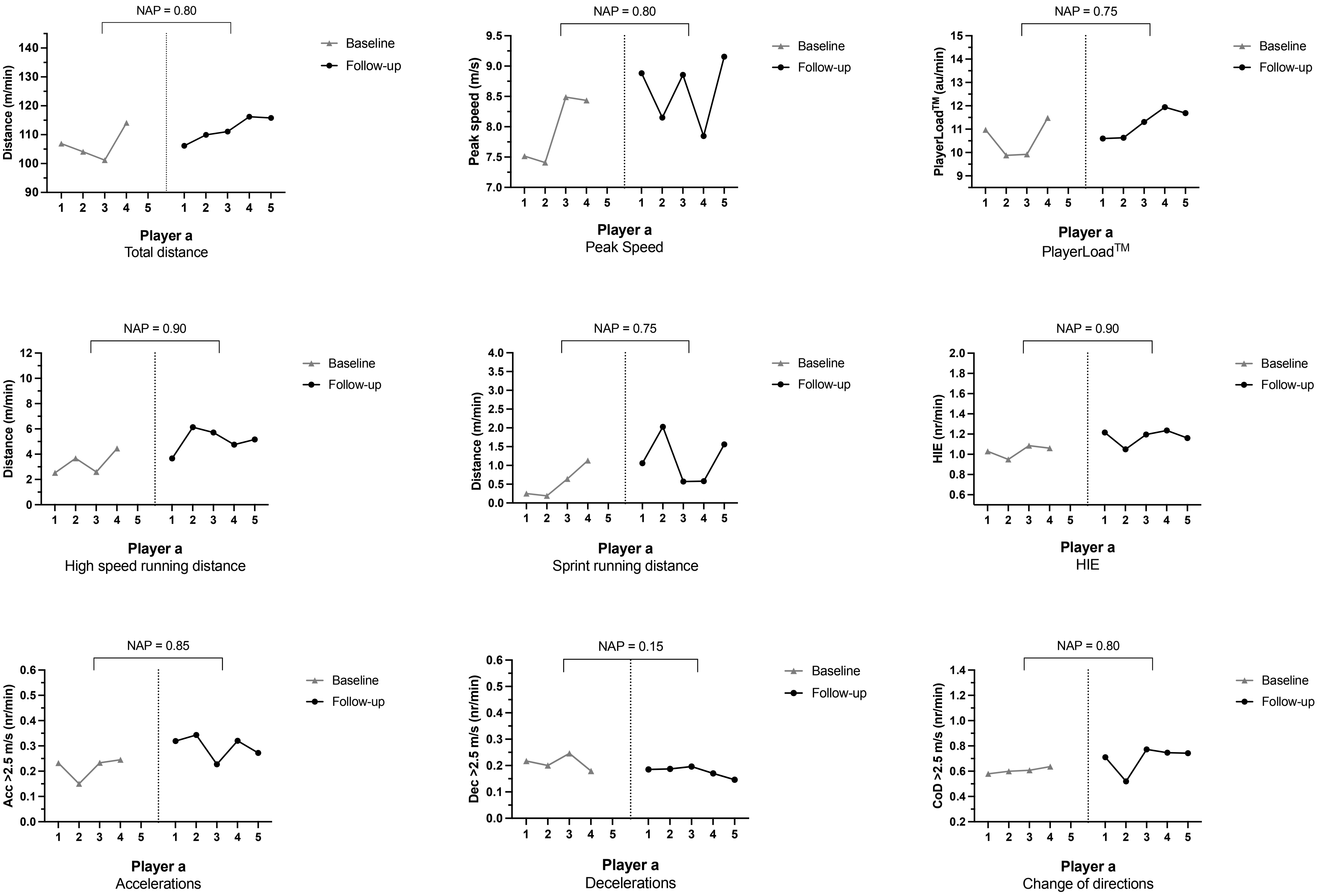
NAP = 0.80
Distance (m/min)
Baseline
Follow-up
Player a
Total distance
NAP = 0.80
Peak speed (m/s)
Baseline
Follow-up
Player a
Peak Speed
NAP = 0.75
PlayerLoadTM (au/min)
Baseline
Follow-up
Player a
PlayerLoadTM
NAP = 0.90
Distance (m/min)
Baseline
Follow-up
Player a
High speed running distance
NAP = 0.75
Distance (m/min)
Baseline
Follow-up
Player a
Sprint running distance
NAP = 0.90
HIE (nr/min)
Baseline
Follow-up
Player a
HIE
NAP = 0.85
Acc >2.5 m/s (nr/min)
Baseline
Follow-up
Player a
Accelerations
NAP = 0.15
Dec >2.5 m/s (nr/min)
Baseline
Follow-up
Player a
Decelerations
NAP = 0.80
CoD >2.5 m/s (nr/min)
Baseline
Follow-up
Player a
Change of directions

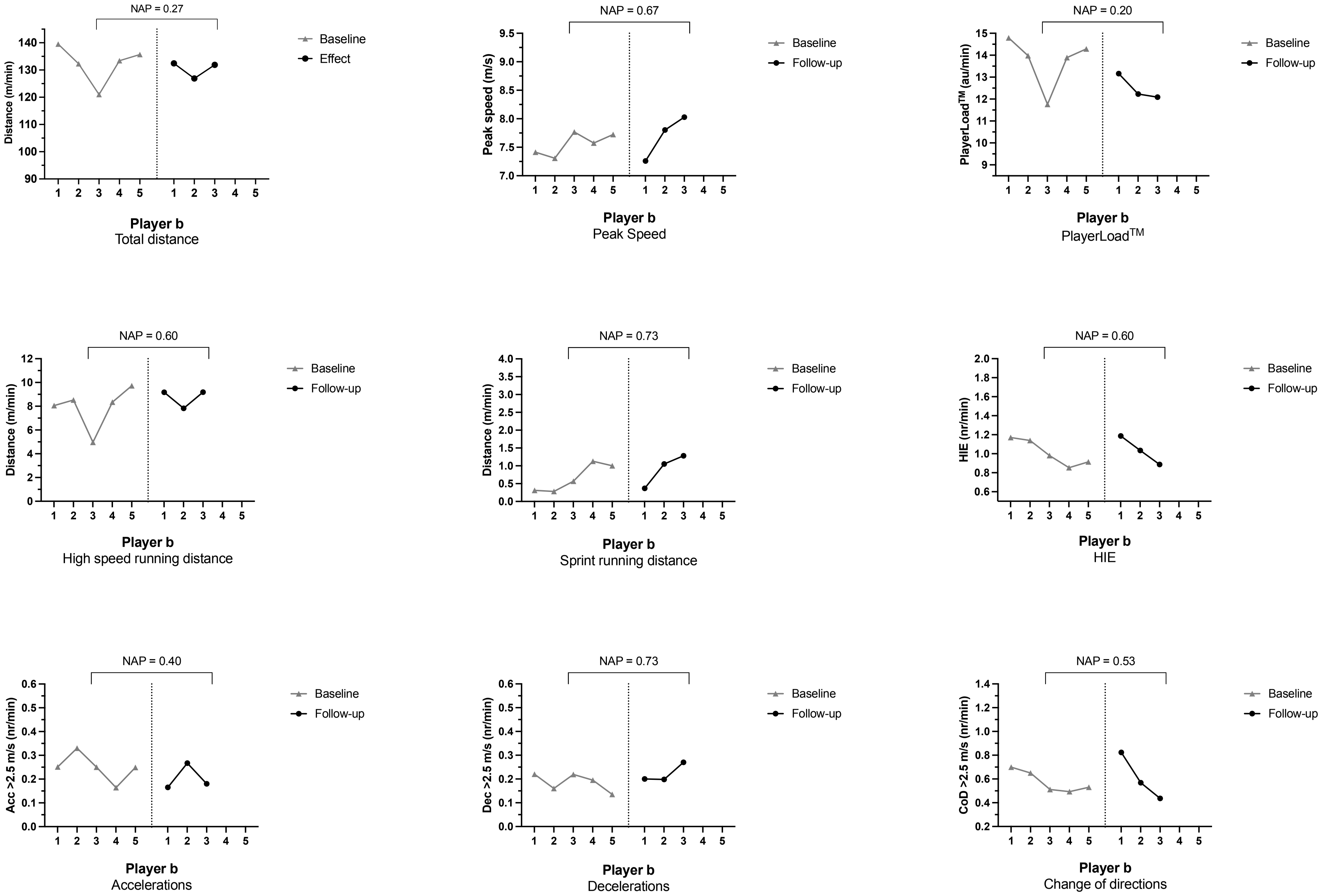
NAP = 0.27
Distance (m/min)
Baseline
Effect
Player b
Total distance
NAP = 0.67
Peak speed (m/s)
Baseline
Follow-up
Player b
Peak Speed
NAP = 0.20
PlayerLoadTM (au/min)
Baseline
Follow-up
Player b
PlayerLoadTM
NAP = 0.60
Distance (m/min)
Baseline
Follow-up
Player b
High speed running distance
NAP = 0.73
Distance (m/min)
Baseline
Follow-up
Player b
Sprint running distance
NAP = 0.60
HIE (nr/min)
Baseline
Follow-up
Player b
HIE
NAP = 0.40
Acc >2.5 m/s (nr/min)
Baseline
Follow-up
Player b
Accelerations
NAP = 0.73
Dec >2.5 m/s (nr/min)
Baseline
Follow-up
Player b
Decelerations
NAP = 0.53
CoD >2.5 m/s (nr/min)
Baseline
Follow-up
Player b
Change of directions

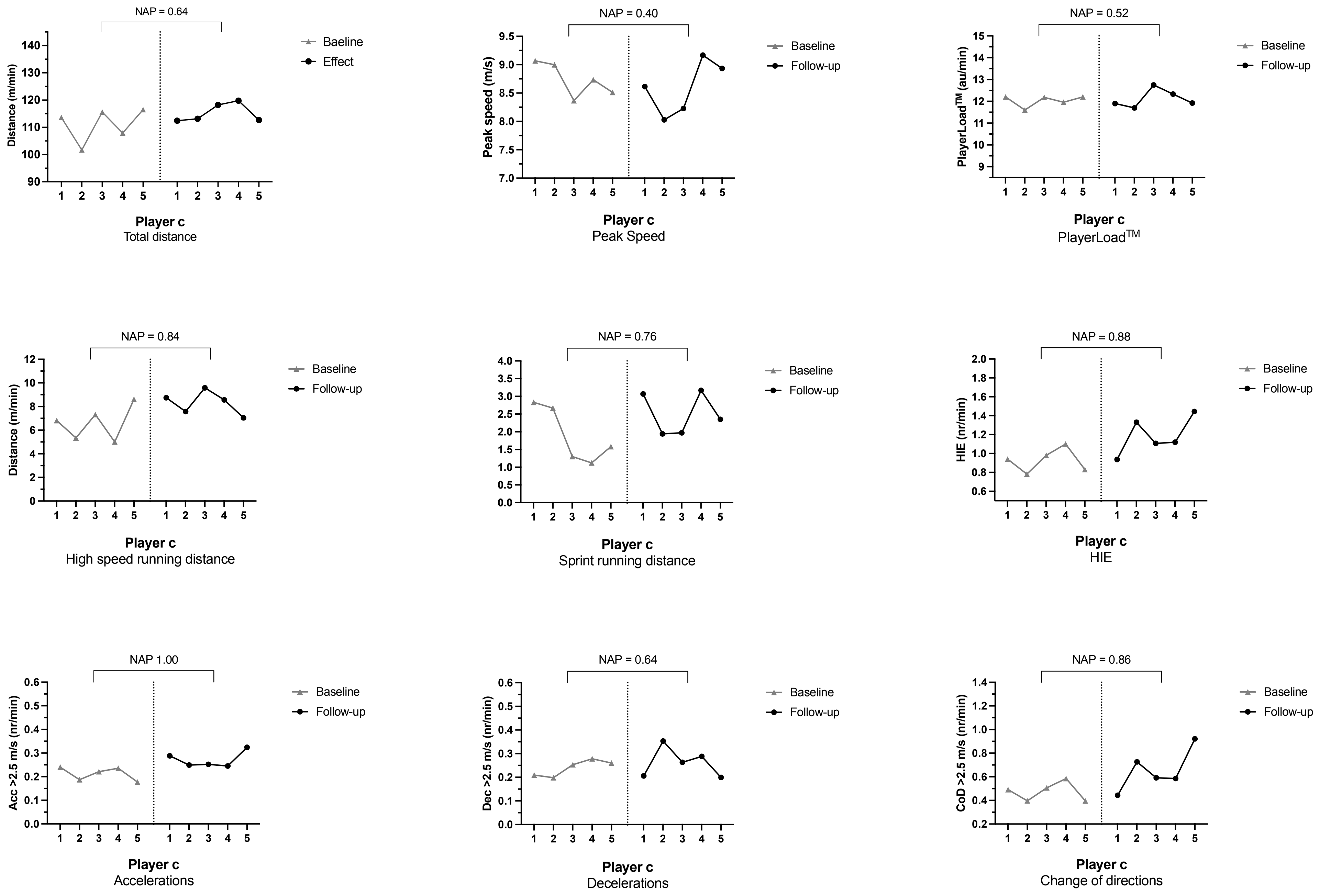

NAP = 0.64
Distance (m/min)
Baeline
Effect
Player c
Total distance
NAP = 0.40
Peak speed (m/s)
Baseline
Follow-up
Player c
Peak Speed
NAP = 0.52
PlayerLoad^{TM} (au/min)
Baseline
Follow-up
Player c
PlayerLoad^{TM}
NAP = 0.84
Distance (m/min)
Baseline
Follow-up
Player c
High speed running distance
NAP = 0.76
Baseline
Follow-up
Player c
Sprint running distance
NAP = 0.88
HIE (nr/min)
Baseline
Follow-up
Player c
HIE
NAP 1.00
Acc >2.5 m/s (nr/min)
Baseline
Follow-up
Player c
Accelerations
NAP = 0.64
Dec >2.5 m/s (nr/min)
Baseline
Follow-up
Player c
Decelerations
NAP = 0.86
CoD >2.5 m/s (nr/min)
Baseline
Follow-up
Player c
Change of directions

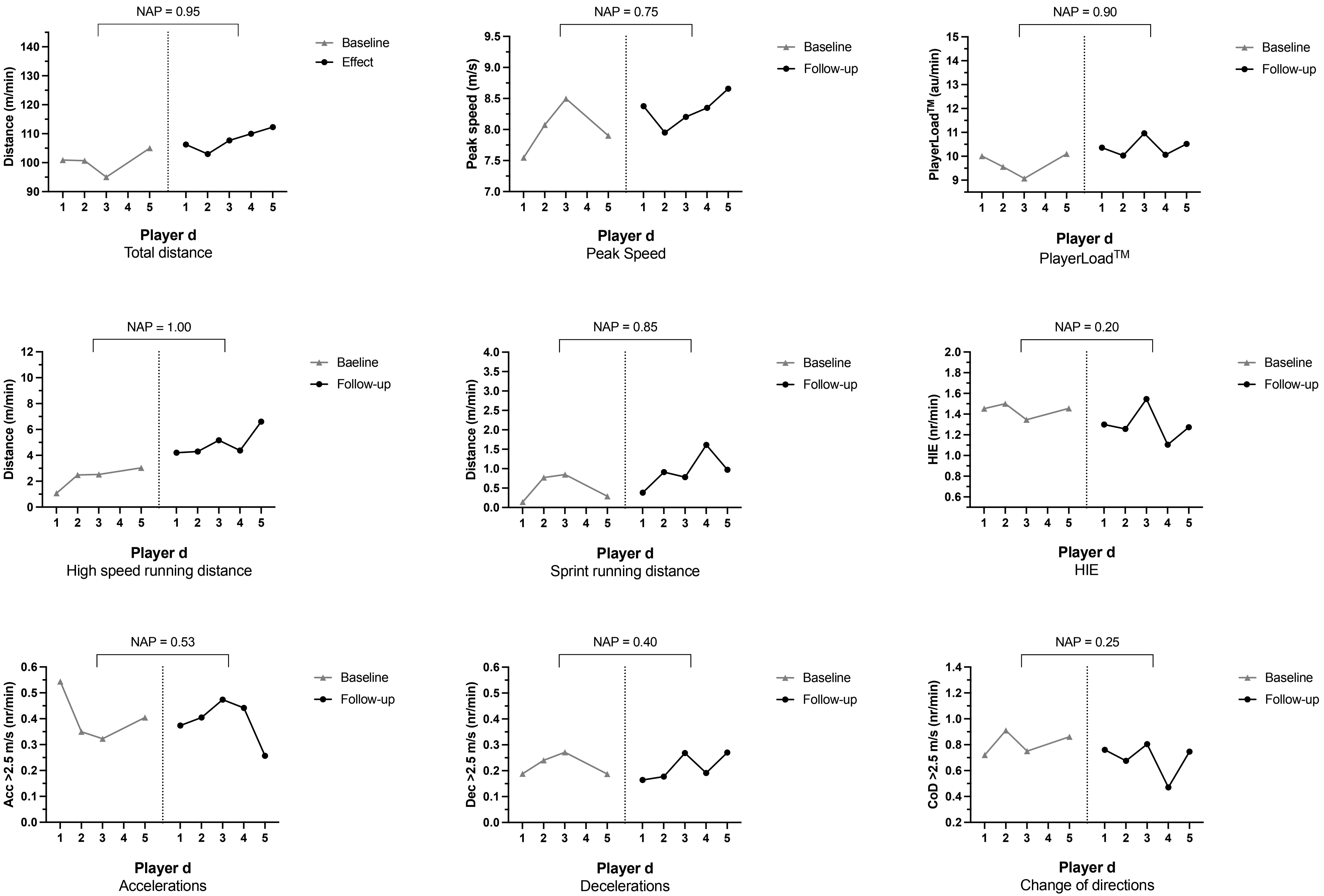
NAP = 0.95
Distance (m/min)
Baseline
Effect
Player d
Total distance
NAP = 0.75
Peak speed (m/s)
Baseline
Follow-up
Player d
Peak Speed
NAP = 0.90
PlayerLoad™ (au/min)
Baseline
Follow-up
Player d
PlayerLoad™
NAP = 1.00
Distance (m/min)
Baeline
Follow-up
Player d
High speed running distance
NAP = 0.85
Distance (m/min)
Baseline
Follow-up
Player d
Sprint running distance
NAP = 0.20
HIE (nr/min)
Baseline
Follow-up
Player d
HIE
NAP = 0.53
Acc >2.5 m/s (nr/min)
Baseline
Follow-up
Player d
Accelerations
NAP = 0.40
Dec >2.5 m/s (nr/min)
Baseline
Follow-up
Player d
Decelerations
NAP = 0.25
CoD >2.5 m/s (nr/min)
Baseline
Follow-up
Player d
Change of directions

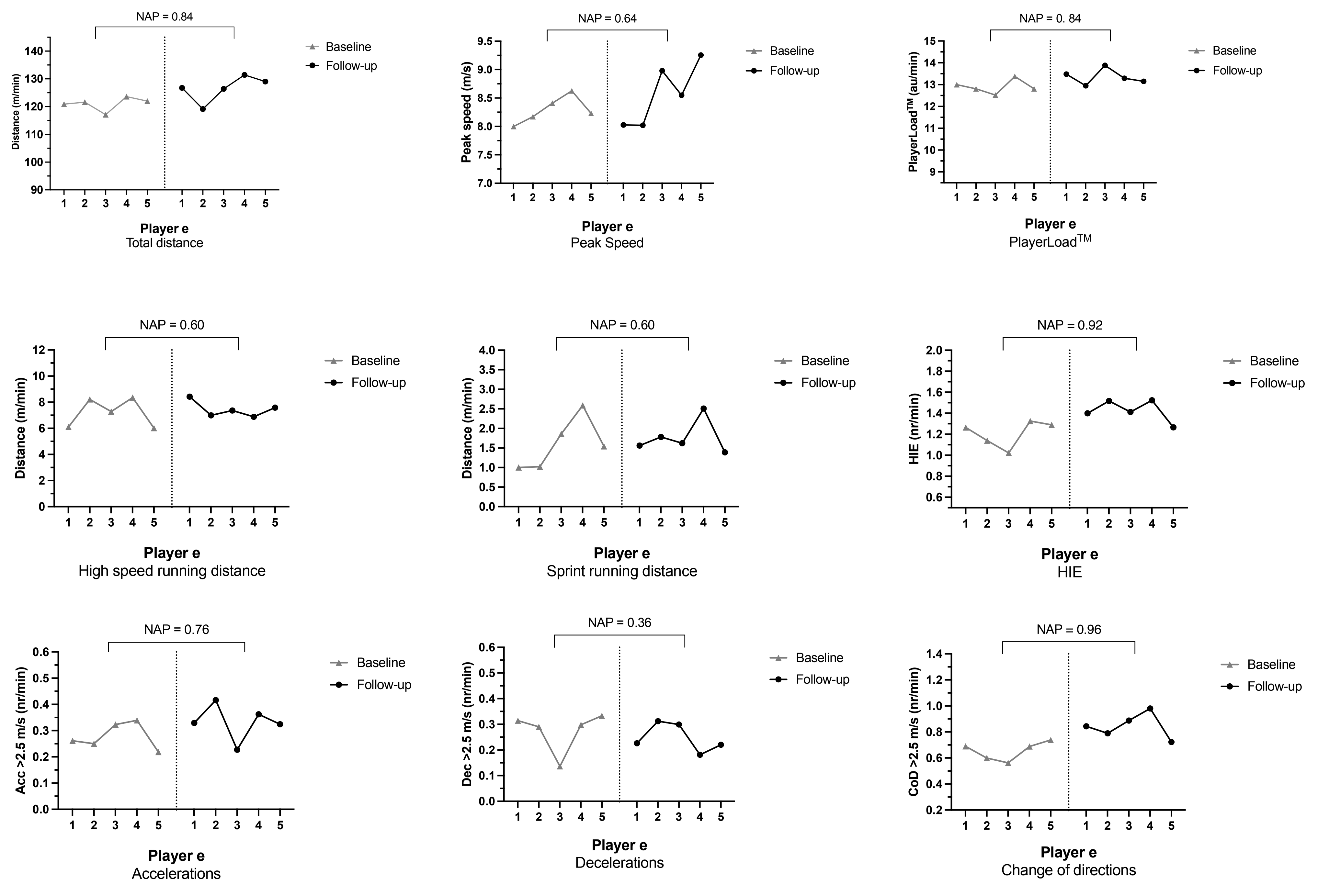
NAP = 0.84
Distance (m/min)
Baseline
Follow-up
Player e
Total distance
NAP = 0.64
Peak speed (m/s)
Player e
Peak Speed
NAP = 0. 84
PlayerLoad^{TM} (au/min)
Player e
PlayerLoad^{TM}
NAP = 0.60
Distance (m/min)
Player e
High speed running distance
NAP = 0.60
Distance (m/min)
Player e
Sprint running distance
NAP = 0.92
HIE (nr/min)
Player e
HIE
NAP = 0.76
Acc >2.5 m/s (nr/min)
Player e
Accelerations
NAP = 0.36
Dec >2.5 m/s (nr/min)
Player e
Decelerations
NAP = 0.96
CoD >2.5 m/s (nr/min)
Player e
Change of directions

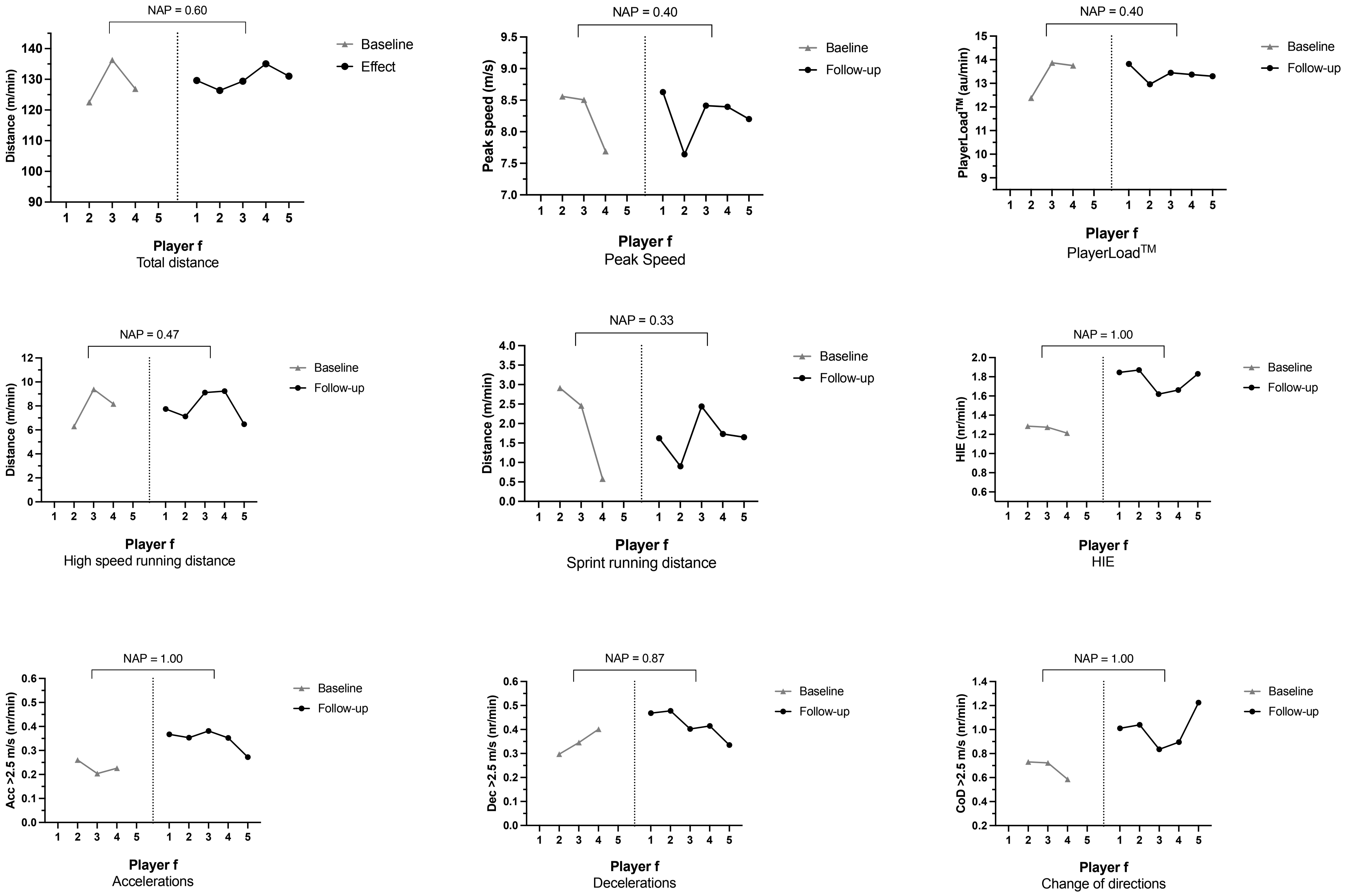
NAP = 0.60
Distance (m/min)
Baseline
Effect
Player f
Total distance
NAP = 0.40
Peak speed (m/s)
Baeline
Follow-up
Player f
Peak Speed
NAP = 0.40
PlayerLoadTM (au/min)
Baseline
Follow-up
Player f
PlayerLoadTM
NAP = 0.47
Distance (m/min)
Baseline
Follow-up
Player f
High speed running distance
NAP = 0.33
Distance (m/min)
Baseline
Follow-up
Player f
Sprint running distance
NAP = 1.00
HIE (nr/min)
Baseline
Follow-up
Player f
HIE
NAP = 1.00
Acc >2.5 m/s (nr/min)
Baseline
Follow-up
Player f
Accelerations
NAP = 0.87
Dec >2.5 m/s (nr/min)
Baseline
Follow-up
Player f
Decelerations
NAP = 1.00
CoD >2.5 m/s (nr/min)
Baseline
Follow-up
Player f
Change of directions

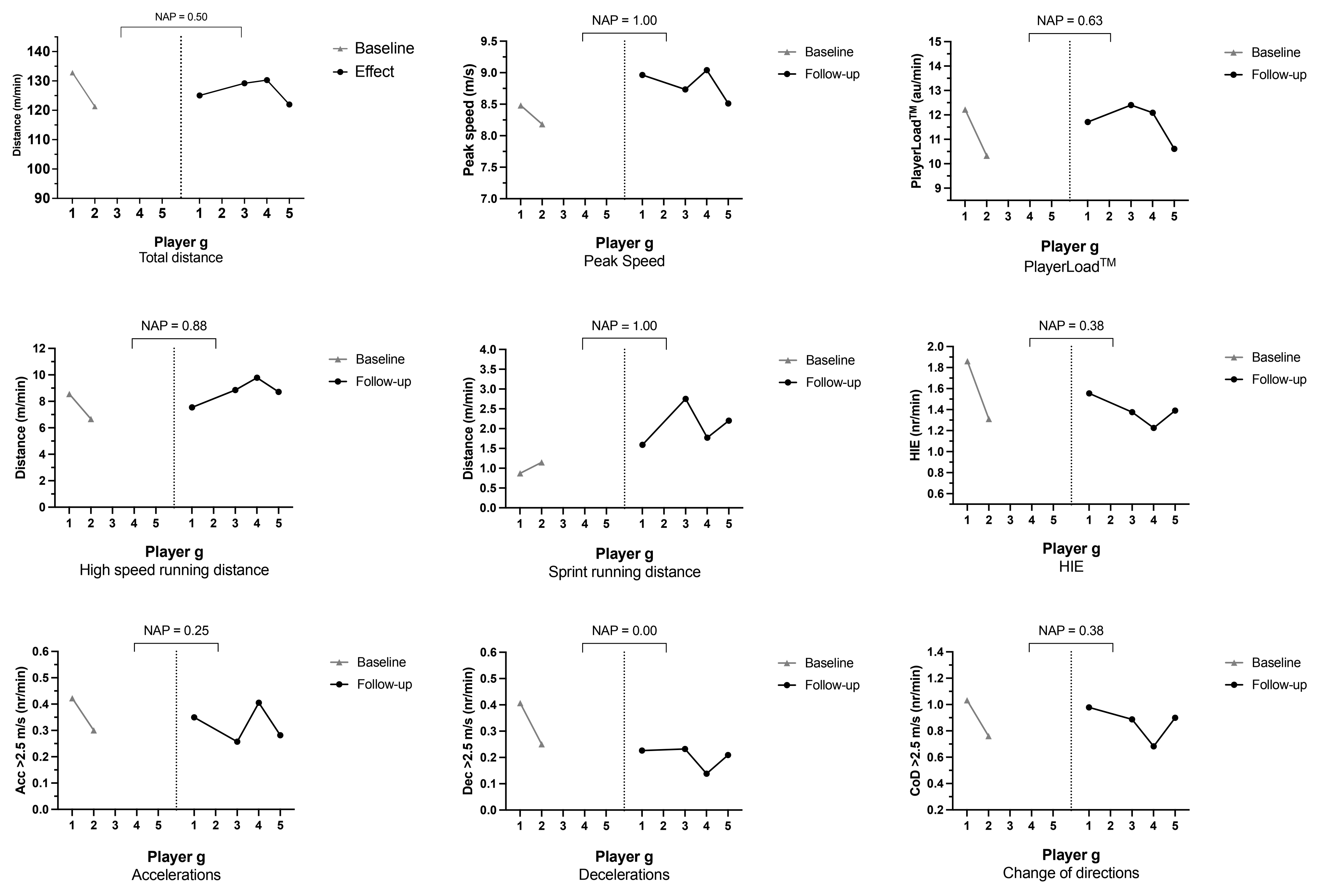

NAP = 0.50
Distance (m/min)
Baseline
Effect
Player g
Total distance
NAP = 1.00
Peak speed (m/s)
Baseline
Follow-up
Player g
Peak Speed
NAP = 0.63
PlayerLoadTM (au/min)
Baseline
Follow-up
Player g
PlayerLoadTM
NAP = 0.88
Distance (m/min)
Baseline
Follow-up
Player g
High speed running distance
NAP = 1.00
Distance (m/min)
Baseline
Follow-up
Player g
Sprint running distance
NAP = 0.38
HIE (nr/min)
Baseline
Follow-up
Player g
HIE
NAP = 0.25
Acc >2.5 m/s (nr/min)
Baseline
Follow-up
Player g
Accelerations
NAP = 0.00
Dec >2.5 m/s (nr/min)
Baseline
Follow-up
Player g
Decelerations
NAP = 0.38
CoD >2.5 m/s (nr/min)
Baseline
Follow-up
Player g
Change of directions

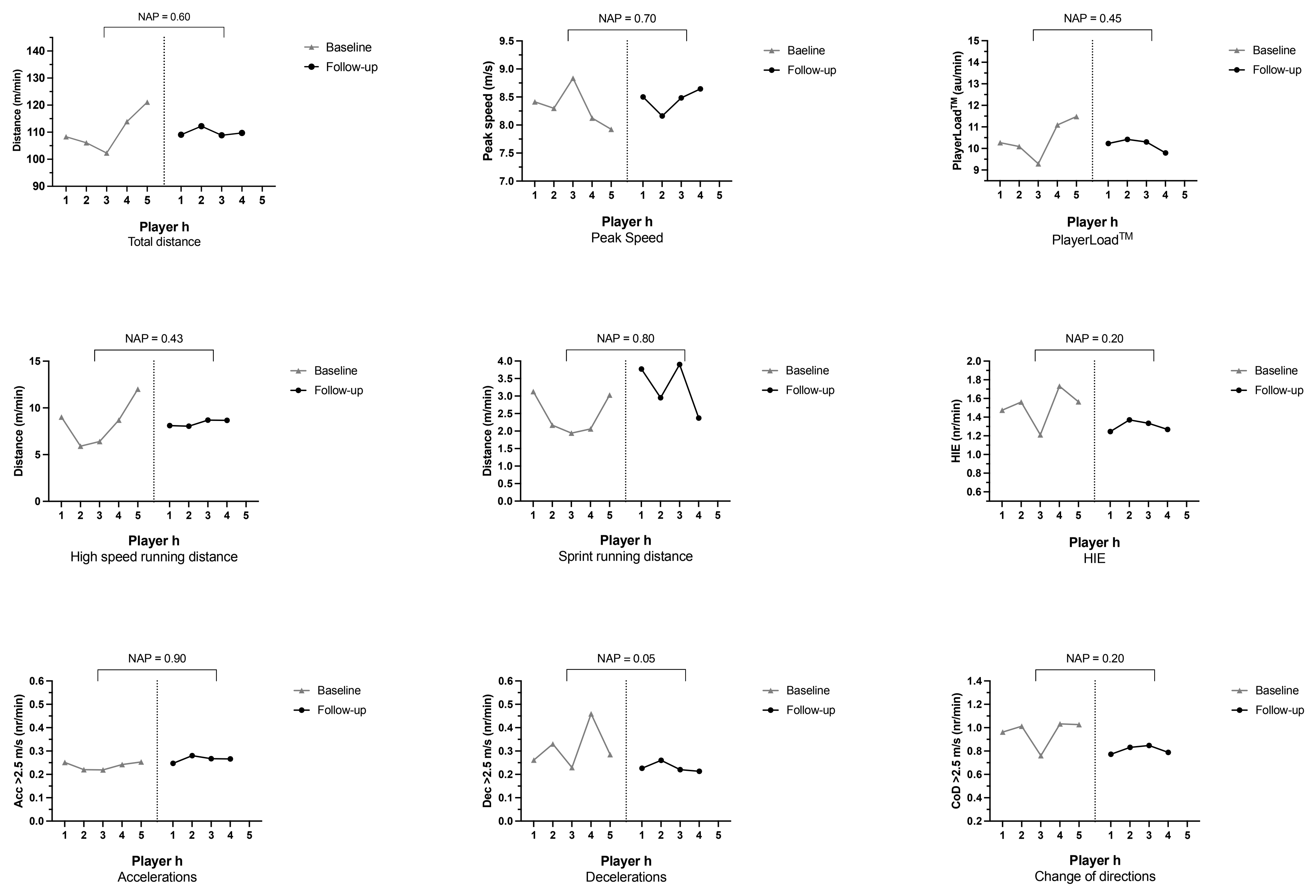
NAP = 0.60
Distance (m/min)
Baseline
Follow-up
Player h
Total distance
NAP = 0.70
Peak speed (m/s)
Baeline
Follow-up
Player h
Peak Speed
NAP = 0.45
PlayerLoad™ (au/min)
Baseline
Follow-up
Player h
PlayerLoad™
NAP = 0.43
Distance (m/min)
Baseline
Follow-up
Player h
High speed running distance
NAP = 0.80
Distance (m/min)
Baseline
Follow-up
Player h
Sprint running distance
NAP = 0.20
HIE (nr/min)
Baseline
Follow-up
Player h
HIE
NAP = 0.90
Acc >2.5 m/s (nr/min)
Baseline
Follow-up
Player h
Accelerations
NAP = 0.05
Dec >2.5 m/s (nr/min)
Baseline
Follow-up
Player h
Decelerations
NAP = 0.20
CoD >2.5 m/s (nr/min)
Baseline
Follow-up
Player h
Change of directions
